# Study on Heat Transfer Characteristics of Graphene Nanofluids in Mini-Channels of Thermal Integrated Building

**DOI:** 10.3390/e25050712

**Published:** 2023-04-25

**Authors:** Yongbin Cui, Dong Liu, Yu Shu

**Affiliations:** 1 School of Urban and Rural Planning and Construction, Mianyang Teachers’ College, Mianyang 621010, China; 2 School of Civil Engineering and Architecture, Southwest University of Science and Technology, Mianyang 621010, China

**Keywords:** graphene, nanofluids, enchanced heat transfer, mini-channels

## Abstract

Two kinds of rectangular mini-channels of different sizes were designed and fabricated for testing the convective heat transfer characteristics of graphene nanofluids. The experimental results show that the average wall temperature decreases with the increases in graphene concentration and Re number at the same heating power. Within the experimental Re number range, the average wall temperature of 0.03% graphene nanofluids in the same rectangular channel decreases by 16% compared with that of water. At the same heating power, the convective heat transfer coefficient increases with the increase in the Re number. The average heat transfer coefficient of water can be increased by 46.7% when the mass concentration of graphene nanofluids is 0.03% and the rib-to-rib ratio is 1:2. In order to better predict the convection heat transfer characteristics of graphene nanofluids in small rectangular channels of different sizes, the convection heat transfer equations applicable to graphene nanofluids of different concentrations in small rectangular channels with different channel rib ratios were fitted, based on factors such as flow Re number, graphene concentration, channel rib ratio, Pr number, and Pe number; the average relative error (MRE) was 8.2%. The mean relative error (MRE) was 8.2%. The equations can thus describe the heat transfer characteristics of graphene nanofluids in rectangular channels with different groove-to-rib ratios.

## 1. Introduction

Mini-channels are widely used in photovoltaic, photo-thermal integrated buildings due to their efficient heat transfer characteristics. In order to improve the efficiency of energy conversion, transportation, distribution, and consumption in integrated buildings, researchers have carried out a great deal of research work in the fields of equipment research and development, energy transfer, the heat exchange working medium, and other aspects. In terms of energy transfer, water is often used as a refrigerant because of its large specific heat and because it is relatively easy to obtain in the current projects, while in terms of heat transfer, it is particularly important to improve the efficiency of fluorine–water or water–air heat exchange.

The method of adding solid particles to water to enhance its heat transfer characteristics has also been generally recognized. Choi and Eastman [1] added nano-sized particles to water and found that this can significantly improve its heat transfer performance, putting forward the concept of nano-fluids for the first time. Azari et al. [2] experimentally studied the flow and heat transfer characteristics of Al_2_O_3_ and TiO_2_ nanofluids in a constant heat-flowing straight circular tube, and found that the heat transfer coefficient increased to 21% and 12%, respectively, after adding Al_2_O_3_ and TiO_2_ nanoparticles. Zeinali Heris et al. [3] studied the heat transfer coefficient of the Al_2_O_3_ nanofluid in a square pipe, and found that its heat transfer coefficient increased with the increase in Reynolds number; the higher flow rate made the nanofluid more uniform, thus strengthening the heat transfer characteristics. Saeed and Kim. [4] studied the heat transfer enhancement characteristics when using nano-fluid Al_2_O_3_-H_2_O as a coolant in the microchannel radiator. The results show that compared with distilled water, nano-fluid significantly improves the convective heat transfer coefficient. At the same time, it can be seen that the prediction of the two-phase mixture model is very consistent with the experimental model, while the convective heat transfer coefficient of the single-phase numerical model is lower than the predicted value. He et al. [5], Kayhani et al. [6], and Rayatzadeh et al. [7] found in flow and heat transfer experimental studies of TiO_2_ nanofluids that the viscosity of nanofluids increases with an increase in particle concentration and size. Given the Re and particle size, the heat transfer coefficient of the fluid increases with an increase in solution concentration, whether in laminar flow or turbulent flow, and the Nu of the fluid increases significantly after ultrasonic oscillation of the solution. However, with a change in Re, Nu has no significant change. Insiat Islam et al. [8] studied the laminar convective heat transfer characteristics of mixed nanofluids in corrugated rectangular tubes with different non-uniform walls. The results show that non-uniform wall waviness has better heat transfer performance than uniform waviness. Buschmann et al. [9] summarized the convective heat transfer characteristics of nanofluids in pipes, pipes with twisted bands inserted, annular countercurrent heat exchangers, and coil and plate heat exchangers. It was found that all nanofluids can be characterized by the Nussel number correlation with water. They showed that the thermal enhancement of nanofluids is related to the thermal conductivity of nanofluids and the movement of nanoparticles, but not to the concentration of nanoparticles or materials. The performance characteristics of heat transfer of nanofluids have been well-summarized in studies by Ganvir et al., Sajid and Ali [10,11], etc.

With the continuous development of graphene technology, researchers also added graphene nanoparticles into the pipeline to study the flow and heat transfer characteristics. Bahiraei et al. [12] compared the differences in flow and heat transfer between graphene nanofluids and other nanofluids. Nagash et al. [13] experimentally studied the flow and heat transfer characteristics of graphene nanofluids in a circular tube with an inner diameter of 11 mm and a length of 109 cm, and concluded that the flow and heat transfer intensity increased with the increase in temperature, Reynolds number and the mass concentration of graphene. The flow and heat transfer intensity of a graphene nanofluid with 0.1% mass concentration was 34% higher than that of the base fluid. Yarmand et al. [14] studied the heat transfer performance of graphene nano-plate (GNP)–platinum (Pt) mixed nanofluids. The experimental results show that all nano-fluid samples have higher heat transfer capacity than the base liquid; Nusselt can increase by 28.48% at most. Fujimoto et al. [15] proposed a new production method for graphene nanofluids and carried out experimental and numerical studies on the turbulent heat transfer performance of graphene nanofluids in horizontal circular tubes with constant heat flux. The results show that the Nu number of a graphene nanofluid produced by pulsed discharge is 33% higher than that of water. Baby and Ramaprabhu [16] prepared graphene nanofluids of different concentrations, with deionized water and ethanol as the base fluid. Through a heat transfer experiment with circular tubes, it was found that the graphene nanofluids using deionized water as the base fluid in the inlet section of circular tubes with an inner diameter of 23 mm and a length of 108 cm increased the convective heat transfer intensity by 64% when the volume concentration was 0.005%, increased the convective heat transfer intensity by 21% at the outlet section, and increased the convective heat transfer intensity by 76% when the volume concentration was 0.01%, where the export segment increased by 57%. Mehrali et al. [17] prepared graphene nanofluids in an eco-friendly way and tested the thermophysical properties of the generated nanofluids. It was found that their thermal conductivity increased significantly. At the same time, the laminar convection heat transfer coefficient of nanofluids under uniform heat flow was tested, which officially confirmed that they have greater heat transfer enhancement ability. Demirkir and Erturk [18] prepared graphene water nanofluids with different mass fractions and experimentally studied their convective heat transfer and flow behavior. It was found that the transition from laminar flow to turbulent flow changed to a lower Reynolds number with the increase in nanoparticle concentration. The Reynolds number of water is 2475, while that of a nanofluid with 0.2% particle mass fraction is 2315. At the same time, due to the influence of thermophoresis and Brownian motion, the heat transfer enhancement in turbulent flow is better than that in laminar flow. When the Reynolds number is 3950, the maximum heat transfer enhancement is 36%. Selvam et al. [19] also reported the same phenomenon and found that although the pressure drop in the turbulent region increased, it was more conducive to the heat transfer process. Balaji et al. [20,21] studied the heat transfer characteristics of functional fossil graphene nanoparticles in the electronic radiator and found that in an experiment using nanofluids based on functional fossil graphene nanoparticles in the radiator, this reduced the radiator temperature by 10 °C and increased the convective heat transfer coefficient and Nussel number by 71% and 60%. At the same time, the application of a water-based mixed nano-fluid with a ratio of graphene nano-plate and multi-walled carbon nanotubes of 1:1 in the radiator can reduce the radiator temperature by 12 °C, and it is considered likely that graphene nano-fluid is an effective coolant to replace conventional coolants for electronic cooling applications. Wang et al. [22] have carried out a numerical study on the flow and heat transfer characteristics in finned annular ducts with different pore numbers and structures. The results show that the eight-hole annular duct has the best performance. The graphene-water nanofluid increases the heat transfer coefficient of a curved finned duct with eight holes and a smooth duct by 11.87% and 11.43%, respectively, and the increase in pressure drop can be ignored. Eric C. Okonkwo et al. [23,24,25] used the characteristic variables of nanoparticle volumetric concentration, mass flow rate, and inlet temperatures to analyze the rate of entropy generated in the solar parabolic trough collector and investigated the influence of the Fe_3_O_4_-Al_2_O_3_/water ternary hybrid nanofluid’s temperature, volume concentration, and mixture ratio on specific heat capacity.

It can be seen from the above results that the research reported in the existing literature focuses on the flow and heat transfer characteristics of nanofluids (including graphene nanofluids) in conventional pipes, while research into heat transfer characteristics in small channels is scarce. Meanwhile, due to the high resistance of nanofluids in turbulent flow in small pipelines, they are not suitable for photovoltaic integrated buildings. Therefore, based on the heat transfer requirements of photovoltaic integrated buildings, this paper has built a flow and heat transfer experimental platform and configured a water-based graphene nanofluid to study its laminar flow and heat transfer characteristics in small rectangular channels, providing experimental and theoretical support for graphene nanoflow to enhance heat transfer.

## 2. Experimental Device and Method

### 2.1. Experimental System and Experimental Section

**Figure 1:**
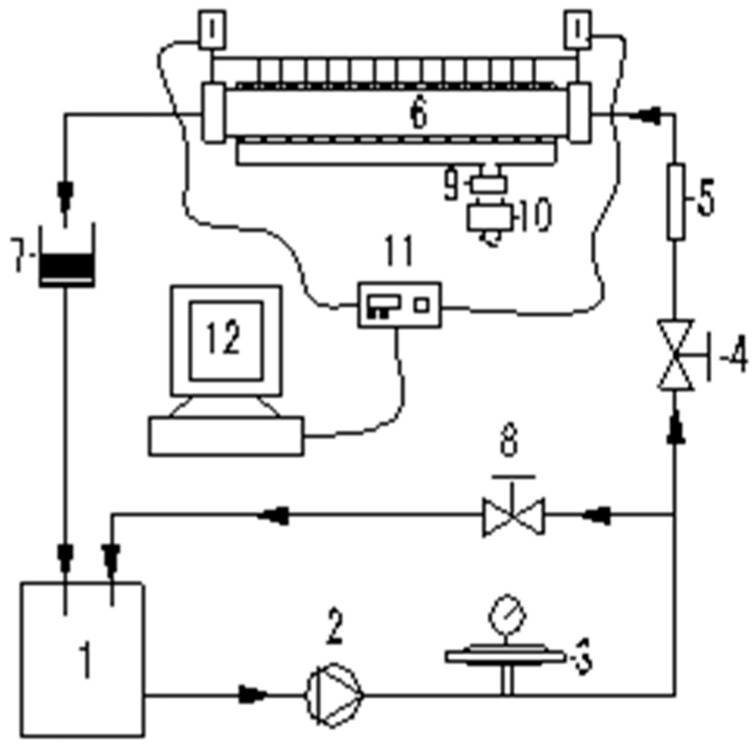
Experimental system diagram: (1) temperature sink, (2) pump, (3) pulsate damper, (4) valve, (5) flowmeter, (6) experimental section, (7) electronic balance, (8) valve, (9) dynamometer, (10) transformer, (11) data collector, (12) computer.

The experimental system is shown in Figure 1. The system is composed of a low-temperature thermostatic bath (1), mechanical diaphragm pump (2), pulsation damper (3), main circulation circuit flow regulating valve (4), rotameter (5), experimental section (6), electronic balance (7), bypass circuit flow regulating valve (8), high-precision power meter (9), transformer (10), data acquisition instrument (11), and data acquisition computer (12). The low-temperature thermostatic bath (1) cools the graphene nano-fluid to the temperature required for the experiment, then it is pumped out by the mechanical diaphragm pump (2). After passing through the pulsation damper (3), the flow control valve (4), and the rotameter (5), it enters the experimental section (6) for heat exchange. It is then mixed with the separated fluid and enters the low-temperature thermostatic bath (1) for cooling and then continues to circulate. In order to accurately control the heating power, this experiment uses a high-precision power meter for adjustment and presents a bottom heater to adjust the heating amount.

**Figure 2:**
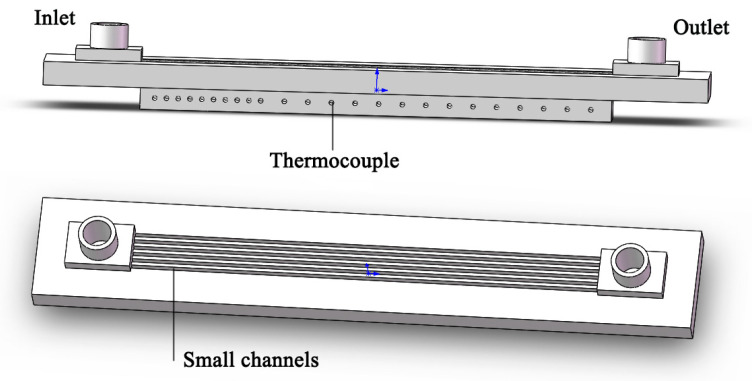
Diagram of the experimental section.

The experimental small rectangular channel heat exchanger is shown in Figure 2. Its external dimensions are 282 mm × 46 mm × 18 mm, the length of the core channel is 200 mm, and the width is 16 mm. It is processed on pure copper by wire-cutting technology (see Table 1 for its geometric parameters). Two rectangular cavities (30 mm long, 16/17 mm wide and 5 mm deep) are designed and processed at the front and rear ends of the inlet and outlet of the small rectangular channel as liquid collecting ports so that the heat exchange medium can flow into the heat exchange channel evenly and stably. In order to measure the wall temperature at the bottom of the channel, a hole with a depth of 8 mm is drilled at a certain interval on the side and 3 mm from the bottom of the channel for the installation of temperature-measuring thermocouples (K-type). The first 10 holes are 5 mm apart and the last 14 holes are 10 mm apart. In addition, a 1-millimeter through-hole is drilled at the inlet and outlet of the liquid collection port to install the temperature thermocouple and measure the fluid temperature at the inlet and outlet.

### 2.2. Preparation of Graphene Nanofluid

The experimental graphene is a reinforced graphene (model SE1430) purchased from Changzhou Sixth Element Material Technology Co., Ltd. (Changzhou, China). Its physical parameters are shown in Table 2.

**Figure 3:**
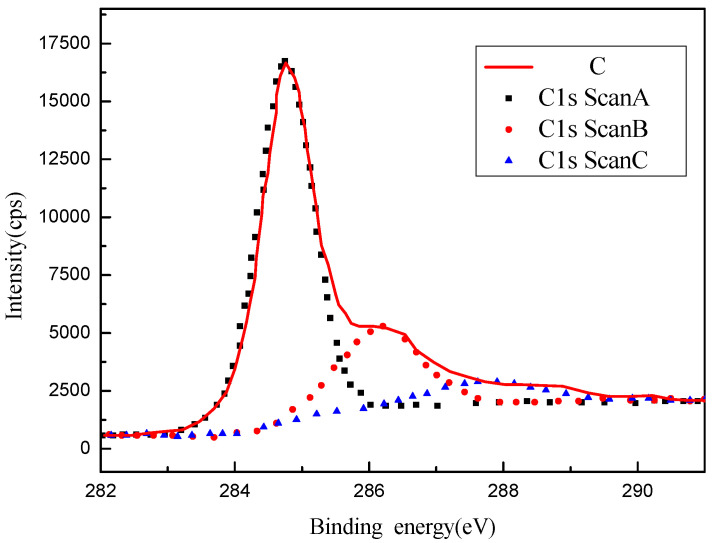
X-ray photoelectron spectroscopy (XPS) patterns of graphene.

**Figure 4:**
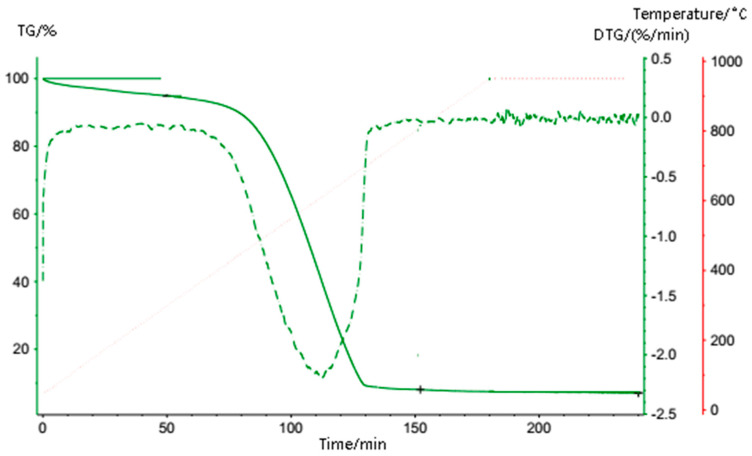
TGA curves of the graphene.

The graphene was also analyzed by X-ray photoelectron spectroscopy (XPS) and thermogravimetric analysis (TGA). The analysis results are shown in Figure 3 and Figure 4, respectively. Table 3 shows the detailed data from the XPS analysis. It can be seen that the peak value of graphene appears at 284.72 eV (C-C bond), which is much stronger than the other peak points of 286.12 eV (C-O-C/C-OH) and 287.72 eV (C=O bond). Therefore, it can be concluded that the oxygen-containing functional groups contained in graphene are less consistent with the general characteristics of graphene. It can be seen from Figure 3 and Figure 4 that the graphene has only a slight mass loss below 450 °C, which is mainly caused by the volatilization of a small amount of water adsorbed on the surface, meaning that the graphene has very good thermal stability below 450 °C.

**Figure 5:**
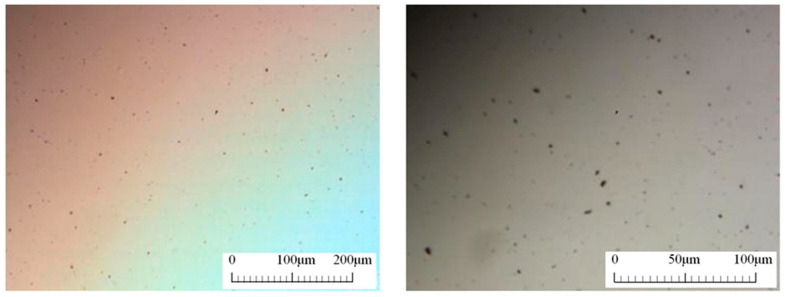
A multi-functional stereomicroscope picture of the graphene nanofluid.

A hydrophilic polymer containing the graphene affinity group was dispersed in deionized water by stirring, then graphene powders of different qualities were weighed. It was added to the base solution containing dispersant and stirred for 1 h by ultrasound to prepare the water-based graphene nanofluid. In order to verify its dispersibility, a simple morphology observation of the solution was carried out through a multi-functional stereomicroscope (Figure 5). It can be seen that the graphene exists uniformly in the solution in a lamellar structure, the transverse size of the particles is 2–3 µm, and there is no agglomeration phenomenon. After sitting for a week, it was found that there was no agglomeration and precipitation phenomenon.

A water-based graphene nano-fluid with strong dispersion stability was prepared and its thermophysical properties were tested by the improved transient plane heat source method in an ambient temperature of 26 °C, using the C-Thermal TCi thermal conductivity instrument (C-Therm Technologies Company, Fredericton, NB, Canada). The thermophysical properties of graphene nanofluids with different proportions were tested (Table 4). In order to ensure the accuracy of the test, each group of data was tested five times and the average value was taken as the final result of each group of data (see Table 4 for the test results). The thermal conductivity (K), specific heat capacity (Cp), heat dissipation rate (e), and viscosity of the solution were found by analyzing the test results (μ); all increase with the concentration.

## 3. Data Processing and Error Analysis

### 3.1. Data Processing

The heat transfer during the flow and heat transfer between the fluid and the small channel wall can be obtained from Formula (1) [26]:

(1) $$Q=mC_{p}(T_{out}-T_{in})$$

where Tin is the inlet temperature of deionized water, in °C; Tout is the outlet temperature of deionized water, in °C; Cp is the specific heat capacity of fluid removal, in kj/(kg·K); m is the mass flow of deionized water, in kg/s.

The channel surface temperature (Tw, x) is calculated by Equation (2) through stable heat conduction. Ttc, x is measured by the thermocouple at 4 mm at the bottom of the channel surface:

(2) $$T_{w,x}=T_{tc,x}-\frac{q_{f}\times H_{tc}}{N\cdot L_{ch}\cdot K_{s}\cdot (w_{ch}+w_{fin})}$$

where Tw, x is the local surface temperature of the channel; Ttc, x is the temperature at the thermocouple measuring point; q_f_ is the fluid heat transfer per unit time; Htc is the distance between the thermocouple and the channel surface and Ks is the channel thermal conductivity; Lch is the channel length; N is the number of channels; Wch and Wfin are channel width and fin width, respectively.

The local fluid temperature along the channel can be calculated by Equation (3):

(3) $$T_{b,x}=T_{in}+\frac{{q^{\prime \prime }}_{conv}\times (2\eta_{fin}H_{ch}+w_{ch})x\times N}{mc_{p}}$$

where T_b, x_ is the local fluid temperature; Tin is the fluid temperature at the inlet; qconv is the heat flow density; η_fin_ is fin efficiency; Hch is channel height; x is the distance from the channel entrance; m is the mass flow rate; c_p_ is the specific heat capacity of the fluid.

The local heat transfer coefficient is calculated by Formula (4):

(4) $$h_{x}=\frac{q_{conv}^{\prime }}{A(T_{w,x}-T_{b,x})}=\frac{q_{f}}{\left(2\eta_{fin}H_{ch}+w_{ch})L_{ch}N(T_{w,x}-T_{b,x}\right)}$$

where $\eta_{fin}$ is the fin efficiency, which can be calculated by Equation (5) [26]:

(5) $$\eta_{fin}=\frac{\tanh(m_{l}H_{ch})}{m_{l}H_{ch}}$$

where m_l_ is the fin parameter, as defined by Formula (6):

(6) $$m_{l}=\sqrt{2\frac{h_{x}}{K_{s}w_{fin}}}\;.$$

The Re number associated with the mixtures of graphene nanofluids can be calculated by the following formula [26]:

(7) Re=uDhν

where u is the mean fluid velocity; D_h_ is the pipe diameter; υ is the graphene nanofluid’s kinematic viscosity.

The average heat transfer coefficient and Nu number can be calculated by the following formula [26]:

(8) $$h_{avg}=\frac{\int h_{x}dA}{A}$$

(9) $$Nu=\frac{h_{avg}D_{h}}{K_{f}}$$

where h_avg_ is the average heat transfer coefficient; A is the channel heat exchange area; D_h_ is the equivalent diameter of the channel; K_f_ is the thermal conductivity of the fluid.

In order to better compare the error of fitting formula and data, the mean relative error (MRE) is defined in Equation (10).

(10) $$MRE=\frac{1}{M}\sum \frac{\left|Nu_{ave,\exp}-Nu_{ave,pred}\right|}{Nu_{ave,\exp}}\times 100\%\;$$

### 3.2. Error Analysis

The temperature-measuring element used in this experiment was a K-type thermocouple with a calibrated accuracy of ±0.4 °C. The flow measuring instrument used was a rotameter, which was calibrated by the mass weighing method before the experiment. The weighing instrument was an electronic balance for the experiment, with an accuracy of ±1 g. The width and height of the channel used in the experimental section were observed and measured by the electronic microscope. Then, the indirect error of these measured data was calculated using error analysis theory; the calculation formula is:

(11) $$\sigma_{y}=\sqrt{{\sum_{i=1}^{n}\left(\frac{\partial f}{\partial x_{i}}\right)}^{2}\sigma^{2}{}_{x_{i}}}\;.$$

The error of each parameter was calculated using Formula (11), and the results are shown in Table 5.

## 4. Results and Discussion

**Figure 6:**
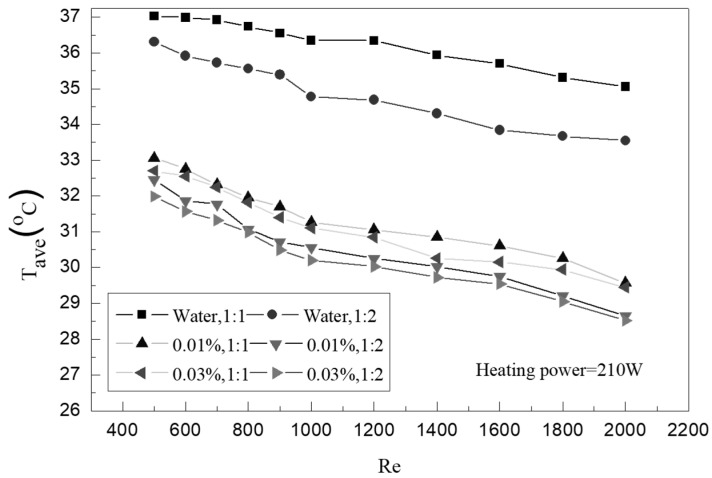
Variation of average wall temperatures with Re.

Figure 6 shows the change in the average wall temperature of the small rectangular channel according to the Re number. It can be seen from the figure that the average wall temperature decreases with the increase in the Re number. At the same Re number, the average wall temperature of the graphene nanofluid is significantly lower than that of water, and that of the small rectangular channel with a rib ratio of 1:2 is better than the channel with a rib ratio of 1:1. When the rib ratio is 1:1, the average wall temperature of the graphene nanofluid at a 0.03% concentration can at most be reduced by 16% compared with water. When the slot rib ratio is 1:2, the maximum reduction can be 14.9%. Therefore, it can be concluded that the graphene nanofluid has an obvious heat transfer enhancement effect when the groove rib ratio is large. By analyzing the results, it can be seen that when the slot rib ratio is large, with the same Re number, the fluid flow rate increases, while under the same power, the fluid temperature rise is low; therefore, the wall temperature decreases. At the same time, when graphene nanofluid is added, the internal mixing of the fluid is strengthened because the internal heat transfer capacity of the fluid is increased; therefore, the wall temperature is lower.

**Figure 7:**
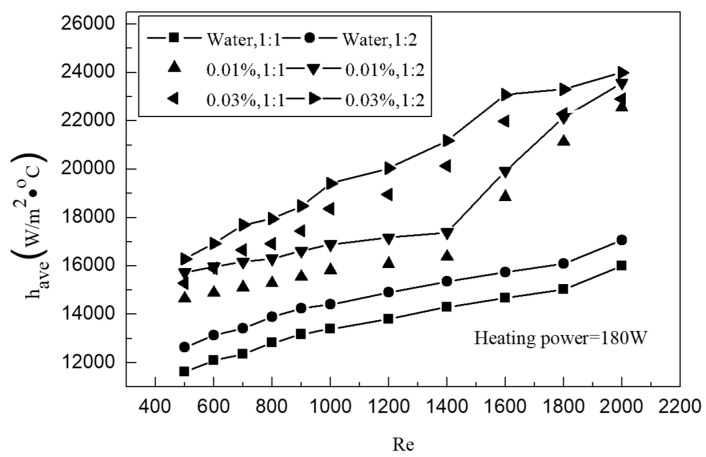
Variation of the average heat transfer coefficient with Re.

**Figure 8:**
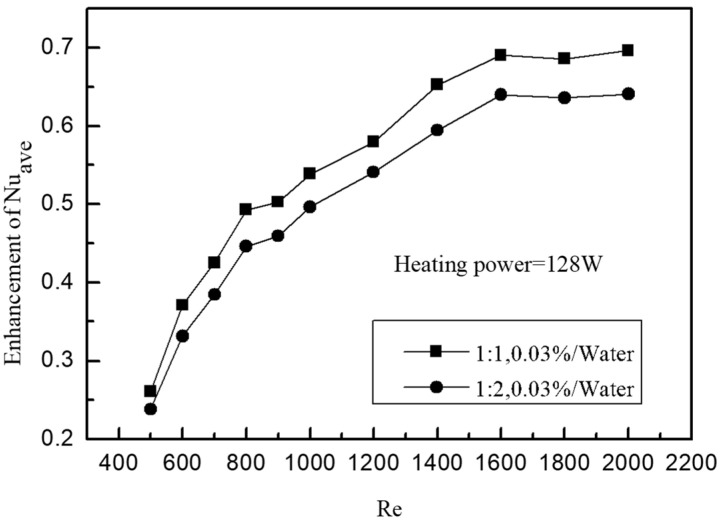
Enhancement of the average Nu.

In order to better reflect the heat transfer characteristics of small rectangular channels, Figure 7 shows the relationship between the average heat transfer coefficient of channels with different proportions and the Re number. It can be seen from the figure that the heat transfer coefficient of solutions with different concentrations in different channels increases with the increase in the Re number. However, the heat transfer characteristics of graphene nanofluids vary with different concentrations. When the Re number is lower than 1400, the average heat transfer coefficient of a 0.03% solution has a faster growth trend than that of a 0.01% solution. When the Re number is higher than 1400, the heat transfer intensity, enhanced by turbulence due to the increase in flow rate in the tank, is far higher than that of graphene nanoparticles. At this time, the enhancement effect caused by different concentrations gradually weakens and finally becomes consistent. When the Re number reaches 2000, the average heat transfer coefficient of solutions at different concentrations is almost equal. On the whole, the average heat transfer coefficient can reach 23,997.2 W/m^2^·°C, which is 46.7% higher than the average heat transfer coefficient of 17,061.4 W/m^2^·°C under the same conditions. In order to better illustrate the enhanced heat transfer characteristics, the average Nu number enhancement ratio is depicted in Figure 8. It can be seen that the 1:1 channel has a higher enhancement ratio than the 1:2 channel, which indicates that the enhanced heat transfer characteristics of graphene nanoparticles in the channel with a low channel–rib ratio are significantly better than that of the channel with a high channel–rib ratio. It is believed that this is the result of the combined effect of the mixed turbulent flow of nanoparticles and the channel size.

**Figure 9:**
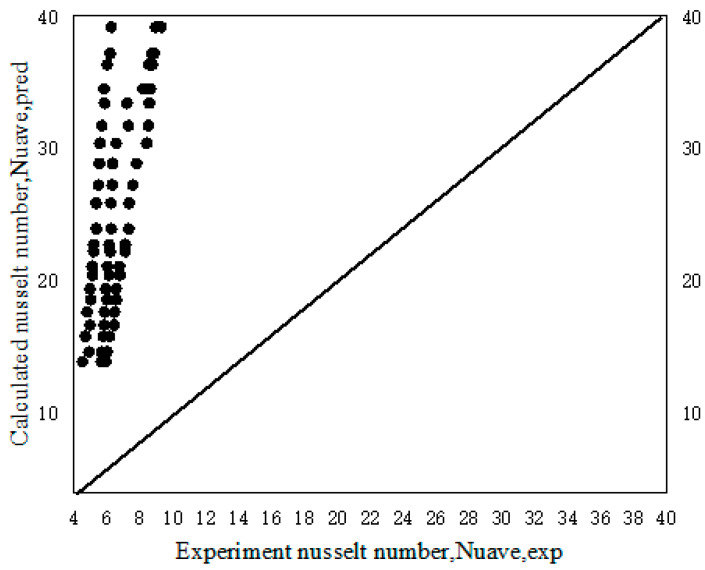
Comparison of the calculated and experimental results of average *Nu.*

We compared the experimental results with the fitting results of Maiga [27]. Figure 9 shows the results comparison chart, with an MRE = 1985.9%, with a large gap. The reason for this may be that in the Maiga experiment, the turbulence in the tube is stronger, the mixing is intense, and the effect of breaking the thermal boundary layer is significant, resulting in its Nusselt number being much higher than that measured in this experiment.

In order to better predict the convective heat transfer intensity of graphene nanofluids with water acting as the heat transfer working medium in small rectangular channels of different sizes, the experimental data were collated and the counter-flow heat transfer equation applicable to graphene nanofluids with different concentrations in small rectangular channels with different channel rib ratios was fitted:

(12) Nu=0.175(1+0.624(φRePe)0.175)Pr1.023WcWf1.143 (Re=500 – 2000)

where φ is the mass concentration of graphene nanofluid; W_c_ is the channel width and Wf is the rib wall width; Pe reflects the proportion of mechanical dispersion and molecular diffusion in the process of dispersion; Pr shows the relationship between the boundary temperature and the flowing boundary layer.

**Figure 10:**
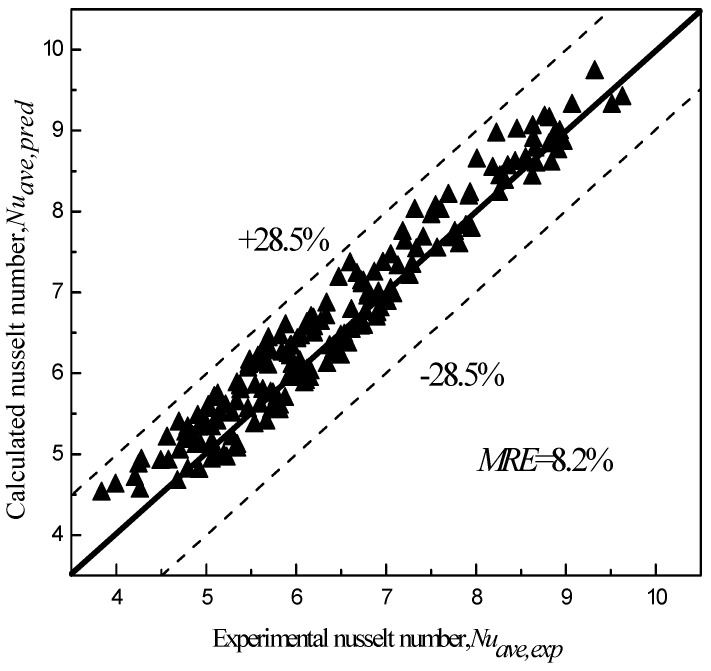
Comparison of the calculated and experimental results of the average Nu.

In order to verify the accuracy of the settlement results of the relationship, the calculation results of the fitting formula were compared with the experimental results, and the average relative error (MRE) was defined to describe its accuracy. From Figure 10, it can be seen that the MRE of the fitting Formula (12) is 8.2% compared with the experimental data. Therefore, the relationship can effectively describe the change in heat transfer intensity of the graphene nanofluid used as the heat exchange working medium in small rectangular channels with different rib ratios.

## 5. Conclusions

In this paper, the flow and heat transfer experimental platform for small rectangular channels with different slot-to-rib ratios was built; water-based graphene nanofluid with different mass concentrations was configured and its flow and heat transfer characteristics in the small rectangular channel were studied, and the following conclusions were obtained:

(1) In the range of the Reynolds number where Re = 500 − 2000, graphene nanofluid shows better heat transfer ability than water. When the mass concentration is 0.03%, the temperature reduction rate of the inner wall of the small channel with a ratio of groove to rib is up to 16%.
(2) The average heat transfer coefficient of the graphene nano-fluid in the small rectangular channel increases with the increase in Re number; the average heat transfer coefficient of the graphene nano-fluid as the heat transfer working medium can increase by 46.7% compared with that of water.
(3) Considering the effects of Re, Pr, height-width ratio, and so on, the relationship that can most accurately describe the convective heat transfer intensity of graphene nanofluid in a small rectangular channel is fitted. The maximum MRE is 8.2%, which can effectively describe the heat transfer characteristics of a graphene nanofluid in a small rectangular channel.

## Figures and Tables

**Table 1:** The structure parameters of the small rectangular channel.

Number	Length of Channel /mm	Height of Channel /mm	Width of Channel /mm	Width of Fin /mm	Number of Channels
#1	200	4	1.5	1.5	6
#2	200	4	1	2	6

**Table 2:** The physical parameters of the experimental graphene.

Model	Exterior	pH	Specific Area (>m^2^/g)	Particle Size (D50, μm)	The Mass Fraction of Carbon (%)
SE1430	Black powder	2.0–5.0	180–280	<10.0	75 ± 5

**Table 3:** The XPS data for the graphene.

Name	Peak BE	FWHM eV	Area (P) CPS.eV	Atomic %	Remark
C1s Scan A	284.72	0.98	16,685.09	70.14	C-C
C1s Scan B	286.12	1.4	5254.77	22.09	C-O-C (C-OH)
C1s Scan C	287.72	1.83	1846.36	7.76	C=O

**Table 4:** The thermophysical properties of graphene nanofluids.

Mass Concentration (%)	Conductivity (W/m·k)	Effusivity (W·s 0.5·m^−2^·K^−1^)	Specific Heat (J/kg·K)	Dynamic Viscosity (mPa·s)
0	0.6101	1587.6	4131.2	1.00
0.01	0.6245	1611.8	4159.9	1.06
0.03	0.6253	1613.3	4162.3	1.23

NOTE: The experimental test temperature is 20–40 °C. Within the experimental testing conditions, the physical parameters of graphene nanofluids show little variation.

**Table 5:** The structure parameters of the small rectangular channel.

Parameter	Max Error/%	Parameter	Max Error/%
*Q*	2.4	h	6.7
T	5.8	D_h_	2.1
Re	7.8	Nu	8.1
